# Research on the care of people with dementia in acute care hospital settings: Opportunities, challenges and limitations

**DOI:** 10.1007/s00391-019-01632-1

**Published:** 2019-10-30

**Authors:** B. Teichmann, J. M. Bauer, K. Beyreuther, A. Kruse

**Affiliations:** 1 Network Aging Research (NAR), Heidelberg University, Bergheimer Straße 20, 69115 Heidelberg, Germany; 2 Bethanien Hospital Heidelberg, Heidelberg, Germany; 3 Institute of Gerontology, Heidelberg University, Heidelberg, Germany

## Introduction

Over 40% of patients over 65 years old in general hospitals show cognitive decline with nearly 1 in 5 suffering from dementia, while dementia is often not detected as a secondary diagnosis at hospital admission [6]. The situation is similar worldwide and will not ease in the next few years due to demographic change, despite a slightly lower incidence of dementia [20, 22].

Due to the heterogeneity of healthcare systems internationally, there is no single solution to improving the situation for people with dementia and hospital staff. To date, 29 of the 194 member states of the World Health Organization (WHO) have adopted their own dementia strategies [3]. In Germany, the participants took up their work in 2019 [8].

Since there are so far no national guidelines for the care of people with dementia (PwD) in Germany, numerous model projects have been carried out that aim to solve specific problems faced by a particular hospital and which were specific for this hospital. Most of these projects have not received scientific support and that is why they are not available for research or for “imitators”.

Research projects can only partially contribute to the nationwide improvement of the care for PwD. Although innovative work has already been carried out, e.g. on delirium prevention, communication with PwD, hospital staff training and transformation into dementia-sensitive hospitals, healthcare organizations often fail to transfer theoretical knowledge into practical skills. There is still a general lack of sustainability and the need for a conceptual framework designed to mandatorily implement established interventions, concepts and guidelines into practice for acute-care hospitals.

## Dementia, symptoms, types and diagnosis

Dementia is the loss of normal cognitive function and behavior (memory, language skills, orientation in time and space, problem solving, self-organization, ability to focus and pay attention) that affects a person’s daily life and activity and may lead to personality changes and the inability to control emotions. Dementia is the result of neuronal dysfunction, neuronal loss and loss of nerve cell contacts. Epigenetic changes in dementia are distinct from normal aging suggesting that dementia disorders are caused by disease processes, whereas normal aging protects from dementia. The causes of dementia depend on changes that occur within the brain. Mixed dementia is very common and is a consequence of the combination of two or more types of dementia. For example, neuropathological studies have shown that up to 80% of patients with diagnosed Alzheimer’s disease (AD) have microinfarcts and atherosclerosis, and approximately 50% have comorbidities including Lewy bodies and transactive response DNA binding protein 43 kDa (TDP-43). These comorbidities may explain the overlapping clinical symptoms between AD and other dementias [7].

## Types of dementia

Several neuropathologies lead to the development of dementia. Major types are AD pathology with neuritic plaques, diffuse plaques, vascular amyloid and neurofibrillary tangles, Lewy body pathology with alpha-synuclein aggregates, hippocampal sclerosis, TDP-43 cytoplasmic inclusions and vascular brain pathologies, such as chronic gross infarcts, moderate to severe arteriolar sclerosis, atherosclerosis and amyloid angiopathy. Recently, it was shown that these neuropathologies are independently associated with lower cognitive levels and cognitive decline [7]. Neuropathological examinations of 1017 study participants who died at age 89.7 (±6.5) years revealed that age-related neuropathologies were present in 94.3% of the participants and frequently comorbid. Of the 94.3% who had at least 1 pathology, 77.8% had 2 or more, 58.2% had 3 or more, 35% had 4 or more, and 16.8% had 5 or more. These comorbidities were found to result in 236 different combinations. The AD neuropathology, not disease, was most commonly observed with 65.3% and identified as pure AD in 9%, but only if observed in isolation. As mentioned up to 80% of patients that fulfilled the diagnostic criteria for AD had vascular comorbidity and 50% showed comorbidity with Lewy bodies and TDP-43 resulting in overlapping clinical symptoms between AD and other dementias [4]. There is a much greater comorbidity of dementia disorders than hitherto appreciated. These comorbidities may not only explain overlapping symptoms but also open new avenues to combat cognitive decline in old people by addressing preventable components, such as cerebrovascular ischemic disease.

## Diagnosis of dementia

Cognitive and neuropsychological tests are mandatory to diagnose dementia by assessing mental functioning, such as memory, language skills, problem solving. These cannot be substituted by laboratory tests (biomarkers) or brain imaging [11]. Laboratory tests and biomarkers help to find causes and exclude other possible disorders. Plasma neurofilament light chain (Nfl) is a very promising noninvasive biomarker to monitor neuronal dysfunction and thus suited for the diagnosis of all types of dementia [16]. Brain imaging is used to identify cerebrovascular ischemic disease, neuronal dysfunction, protein pathologies, e.g. amyloid positron emission tomography (PET) and tau PET, and brain atrophy. Genetic tests help to identify people carrying gene defects that causes early onset dementia but strictly require genetic counselling. Last, psychiatric evaluation can help if depression or other mental health conditions cause or contribute to the symptoms.

## National dementia strategies

Dementia, which predominantly affects older people, has been referred to as a major public health problem due to increasing life expectancy along with the lack of efficient therapeutic strategies [24]. Dementia is one of the major causes of disability and dependency among older people worldwide. According to the World Health Organization (WHO), in 2015, dementia affected 47 million people worldwide, which corresponds to 5% of the world’s older population. This number is predicted to increase to 75 million in 2030 and 132 million in 2050, doubling every 20 years [17]. During the last 10 years, 29 countries already developed their own national dementia strategy (NDS) to provide appropriate care to patients with dementia (PwD) and support to their families and 29 countries have a national dementia plan in preparation [3], with France being the first in Europe to launch a national plan in 2001. Most of them have the objective of increasing awareness, establishing support services, enhancing standard care, improving both training and education for healthcare practitioners, as well as promoting research [10].

In May 2017, the WHO adopted the global action plan on the public health response to dementia 2019–2025 and in 2018 published a WHO guide [28], which provides technical guidance to their member states in creating and operationalizing a dementia plan.

While the priorities of the NDS of each country are common to all, details and concepts differ from country to country. Only a few of the already established NDSs have a focus on PwD in general hospitals and developed a coordinated care pathway for PwD, e.g. the UK [12], to improve quality of care towards the end of life and/or shift the care from hospitals to multidisciplinary, community-based settings. The importance of a global strategy for PwD in general hospitals cannot be overestimated due to the fact that PwD have twice as many hospital stays per year as other older people [2]. It is estimated that one quarter of hospitalized patients have a dementia diagnosis in addition to primary hospital admission diagnosis [1]. One of the major factors which has been shown to affect the care quality in hospital settings is the lack of proper dementia knowledge [14].

The hospital stay is particularly challenging for PwD. Up to 75% will experience behavioral and psychological symptoms of dementia (BPSD) [27] including agitation, aggression, affective disorder, wandering, hallucinations and sleep disturbances [21]. Moreover, the hospital surrounding is unsuitable for PwD and can worsen BPSD [14]. In order to avoid stressful situations for patients, their relatives and the hospital staff, a basic understanding of the disease, BPSD and the possibilities of communication with PwD are necessary. Because of the different focuses of the NDSs, different training programs for hospital staff and different structural conditions, an evaluation and comparison of existing programs is extremely difficult and the needs differ not only from country to country but also within the country due to the autonomous structure of hospitals in some countries. Most of the studies which present structured programs to improve the quality of life of PwD, their relatives and hospital staff come from those countries that started with a NDS early on, such as the UK, Canada and the USA.

## Dementia in hospital patients

The recently published General Hospital Study, which was funded by the Robert Bosch Foundation and the German Alzheimer Society (Deutsche Alzheimer Gesellschaft), demonstrated that the prevalence of dementia and cognitive impairment approximates a prevalence of two in five patients beyond the age of 65 years who are admitted to general hospitals in Germany [6]. Of the participants 18.4% had dementia and 19.8% showed signs of minor cognitive impairment. The highest prevalence rates for PwD were observed among those admitted to departments of internal medicine and trauma surgery. In this cross-sectional study, patients admitted to departments of geriatric medicine, neurology or psychiatry were not included in the analysis, while the prevalence of dementia and cognitive impairment would be expected to be even higher in these disciplines. The German prevalence rates are close to those reported in a recent study from the UK [26]. According to the authors of the German prevalence study, the age distribution of hospital patients contributed to the high prevalence of both conditions as well as the high admission rate of patients affected by these two conditions and the prolonged length of stay observed in this population. The aforementioned prevalence studies highlight one of the great challenges for German healthcare in the context of the demographic shift as routine screening for dementia and delirium as well as adapted care have not yet been implemented on a routine basis.

Hospital care in PwD and delirium has to meet the special needs of this population, as they are affected by a higher rate of complications, functional decline and increased short-term as well as medium-term mortality [18]. In this context, it should be considered that it is not only the hospital environment that causes these negative consequences but that the patients’ overall medical condition is significantly intertwined with it. Nevertheless, it should be a major focus of clinical research in geriatric medicine to develop and validate programs that decrease the prevalence of complications and thus stabilize functionality in this population. One approach could be characterized by specific efforts to decrease the length of stay in patients with dementia and subsequent outpatient care including instructions on adapted physical exercise for this population, e.g. lifestyle integrated functional exercise [5]. Alternatively, the concept of early inpatient rehabilitation, which includes among other components, physiotherapy, occupational therapy, neuropsychology and logopedics and which is widely applied in German geriatric hospital patients, may need modification reflecting the specific needs of PwD and delirium. Possibly both approaches may be valuable in subpopulations of PwD and/or delirium who would be defined by differing comorbidities. The identification of appropriate outcome measures for this population will enable us to take one important step forward in this field of clinical research [19]; however, the needs of PwD and delirium cannot be adequately addressed only by geriatric departments. It will also be necessary to develop programs that can be easily applied both in internal medicine and surgical departments. Guidelines and some successful pilot projects provide first clues to how these requirements can be met [25]; however, additional research is currently still needed on a broad scale. The results of the Robert Bosch postgraduate seminar on dementia in hospitals will be helpful for answering at least some of the urgent questions in this important field of care.

## Promoting participation of people with dementia as a political task

The question remains open whether research alone is sufficient to bring about the necessary nationwide changes. Publications have shown that national strategies that establish a stable conceptual framework can be helpful in implementation of such programs. In the recent coalition agreement of the national government of Germany, it was decided to initiate a NDS, which is currently being developed under the auspices of the Federal Ministry for Family Affairs, Women, Senior Citizens and Youth and the Federal Ministry of Health. A branch office set up at the German Center for Gerontology has the task of supporting the process of developing the NDS in terms of content and organization and four action areas have so far been defined [13]. “Care for people with dementia in hospital” forms the subject of discussions assigned to action area 3 of the national dementia working group. It attaches priority to care in inpatient care facilities and (acute care) hospitals, as the situation of PwD is characterized there by particularly high vulnerability. Accordingly, this working group focuses on (a) avoiding risks being associated with unnecessary hospitalization, (b) improving the special form of hospitalization (e.g. by integrating screening procedures) and (c) making acute hospitals “dementia-sensitive” both spatially and organizationally (e.g. by financial support of conversions, improved medication plans or provision of basic gerontological qualifications).

## Approaches to shared responsibility

As important as the NDS is for a socially responsible discourse, it should also be emphasized that this strategy must be regarded as politically compulsory to an extent that its recommendations on structures of social participation, support for PwD and their relatives, medical and nursing care, basic research and application-based research, are directly incorporated into legislative measures.

The participatory design of public realm is, first and foremost, understood as one of the fundamental tasks of municipality, the municipality as a local authority and as a community of citizens. The municipality as a local authority is responsible for services of general interest (*Daseinsvorsorge*; [15, 23]), which aims to create conditions under which autonomy, participation and quality of life can be developed, maintained and further differentiated. These services of general interest must by no means exclude PwD and their relatives but have to ensure that these services are extended and differentiated in such a way that they are optimally tailored to the needs of PwD and their relatives. As a result, municipalities are particularly challenged to create an infrastructure of care and support services that (also) demonstrates a high degree of responsiveness to the specific vulnerabilities of PwD and their families. An important topic is to what extent such services could be financed through the municipalities alone. Many municipalities cannot afford appropriate resources; for this reason shared responsibility of municipalities, federal states and Federal Government is of high political importance.

## People with dementia from the perspective of impairments

Fostering participation of people with dementia would, in our view, be further promoted by the fact that they are perceived not only as people being in need of care but also as people with disabilities. In accordance with the 2016 Federal Government report on the situation of people with disabilities this would focus on those “concrete restrictions that arise in interaction with the environmental conditions and thus influence the social participation opportunities” [9]. This means that not only strengthening of functions and skills of people with dementia should be strived for but also a change of social, ecological and institutional environments with the aim of contributing to the longest possible preservation of competence and quality of life. In our view, the development of care and support for people with dementia from the perspective of participation is an important element of social policy innovation. Here, the Nursing Strengthening Act (above all PSG II 2016) as well as a correspondingly interpreted Federal Participation Act (BTHG, 2016) would have to be integrated and harmonized. Such integration and harmonization would benefit PwD and their relatives in all phases and forms of care, care, support and promotion also with respect to care in hospitals.
